# Individual, Interpersonal, and Obstetric Factors Associated with Exclusive Breastfeeding Among Polish Mothers: A Cross-Sectional Study Informed by the Socio-Ecological Model

**DOI:** 10.3390/nu18172900

**Published:** 2026-09-04

**Authors:** Agnieszka Czerwińska-Osipiak, Amelia Julia Sobala, Anna Weronika Szablewska, Krzysztof Jurek

**Affiliations:** 1 Department of Obstetric and Gynecological Nursing, Institute of Nursing and Midwifery, Medical University of Gdańsk, M. Skłodowskiej-Curie 3 A, 80-210 Gdansk, Poland; anna.szablewska@gumed.edu.pl; 2 Institute of Sociological Sciences, Faculty of Social Sciences, John Paul II Catholic University of Lublin, Al. Racławickie 14, 20-950 Lublin, Poland; krzysztof.jurek@kul.pl

**Keywords:** breastfeeding, socio-ecological model, birth satisfaction, attitudes towards breastfeeding, partner support, social support, infant

## Abstract

Background/Objectives: Exclusive breastfeeding (EBF) is associated with multiple individual, interpersonal, and healthcare-related factors. Identifying potentially modifiable factors associated with EBF across different levels of influence may help inform future breastfeeding research and support strategies. This study aimed to identify individual, interpersonal, and obstetric factors associated with exclusive breastfeeding among Polish mothers, using the Socio-Ecological Model (SEM) as a conceptual framework. Methods: A cross-sectional online survey was conducted among 769 Polish mothers of children aged up to 24 months. The questionnaire included sociodemographic and perinatal characteristics, as well as standardized instruments: Multidimensional Scale of Perceived Social Support (MSPSS), Iowa Infant Feeding Attitudes Scale (IIFAS), and Birth Satisfaction Scale–Revised (BSS-R). Hierarchical binary logistic regression was used to identify factors associated with exclusive breastfeeding. Missing data were handled using multiple imputation by chained equations, with 20 imputed datasets, and estimates were pooled according to Rubin’s rules. Results: Exclusive breastfeeding was reported by 610 participants (79.3%). Across the 20 imputed datasets, the final model showed *χ*^2^ values ranging from 62.92 to 70.00 and Nagelkerke’s *R*^2^ values ranging from 0.123 to 0.136. In the final model, mode of delivery (OR = 1.92, 95% CI: 1.32–2.80, *p* < 0.001), complications/illnesses during pregnancy (OR = 1.73, 95% CI: 1.14–2.64, *p* = 0.011), and partner encouragement (OR = 2.81, 95% CI: 1.72–4.59, *p* < 0.001) were significantly associated with exclusive breastfeeding. A nonlinear association between breastfeeding attitudes and exclusive breastfeeding was observed, with the quadratic IIFAS term remaining statistically significant (OR = 0.99, 95% CI: 0.99–1.00, *p* = 0.002), whereas the linear IIFAS term was not statistically significant in the final model (OR = 1.04, 95% CI: 1.00–1.08, *p* = 0.078). Birth satisfaction and the MSPSS Friends, Family, and Significant Other subscales were not independently associated with exclusive breastfeeding in the final model. Conclusions: Among the variables included in the final model, partner encouragement showed the strongest association with exclusive breastfeeding. Mode of delivery and complications/illnesses during pregnancy were also associated with exclusive breastfeeding, while the association between breastfeeding attitudes and exclusive breastfeeding was nonlinear. These findings may inform future prospective and intervention studies examining partner support, breastfeeding attitudes, and perinatal factors in relation to exclusive breastfeeding. Further research is warranted to explore the mechanisms underlying the observed associations.

## 1. Introduction

Breastfeeding is the optimal method of infant nutrition, providing numerous health benefits for both the infant and the mother. It has been shown to reduce the risk of infections, hospitalizations, and mortality in infants. Conversely, among breastfeeding mothers, lower incidence rates of breast and ovarian cancer, postpartum depression, and type 2 diabetes have been observed.

The latest reports from the World Health Organization (WHO) and UNICEF (United Nations Children’s Fund) provide a comprehensive picture of the current global breastfeeding landscape. It is estimated that nearly all newborns (approximately 95%) have had at least a single exposure to breast milk. Additionally, the global rate of infants put to the breast within the first hour after birth currently stands at 48%, reflecting a marked improvement compared to previous years. The rate of exclusive breastfeeding during the first six months of life remains at approximately 47%, with significant regional disparities—the highest rates are recorded in South Asia, while the lowest are found in North America [1,2]. Current European data indicate that, despite relatively high breastfeeding initiation rates, sustaining exclusive breastfeeding in the subsequent months of infancy remains challenging. A steady decline in the proportion of exclusively breastfed infants can be observed over time, which results in a low average rate of approximately 25% at six months of age.

The situation in Poland reflects this general pattern. Although the breastfeeding initiation rate is very high, approaching nearly 100%, a gradual decline is noted in the subsequent months of the infant’s life [3,4]. Nevertheless, the most recent national findings from the PITNUTS 2024 study, conducted by the Institute of Mother and Child, indicate an improvement in the situation, with increases observed both in the overall proportion of breastfed children and in the rate of exclusive breastfeeding during the first six months of life. According to report data, 72% of women provide their infants with breast milk, representing substantial progress compared to the 54% recorded in 2016. Furthermore, between the fifth and sixth months of life, the overall proportion of exclusively breastfed infants is 41.0%, indicating a marked increase relative to previous years, when this value remained at approximately 6% [5].

Despite this positive trend, the proportion of mothers adhering to the recommendation of exclusive breastfeeding for the first six months remains limited. In response to the global trend in breastfeeding, the World Health Organization (WHO) has established new global targets, aiming to increase the exclusive breastfeeding rate to 60% by 2030, alongside improving early breastfeeding initiation. Achieving these goals is of critical importance, not only for improving maternal and child health, but also for attaining broader health, economic, and environmental objectives [6]. Despite unequivocal international recommendations advocating for exclusive breastfeeding during the first six months of life, the proportion of women adhering to these guidelines in many countries remains below expected levels, underscoring the need to identify factors associated with lactation behaviors.

Urie Bronfenbrenner’s theory of human development posits that it is the result of reciprocal interactions between an individual and his or her immediate environment, as well as the broader social context. Bronfenbrenner described four fundamental systems influencing individual development: the microsystem, mesosystem, exosystem, and macrosystem. In relation to breastfeeding, particular significance is attributed to the microsystem—that is, the woman’s immediate living environment, encompassing her partner, family, friends, healthcare personnel, workplace, and individuals involved in infant care. Indeed, the daily relationships and experiences within this setting can substantially influence a mother’s decisions regarding the initiation and continuation of breastfeeding.

In the context of health promotion, Bronfenbrenner’s theory has been operationalized within the Socio-Ecological Model, according to which health behaviors are the result of an interplay of multiple factors operating at individual, interpersonal, community, organizational, and policy levels [7]. Given the complex and multifactorial nature of infant feeding behaviors, the analysis of factors associated with breastfeeding can be effectively based on the tenets of the SEM. Decisions regarding breastfeeding are the outcome of interactions among factors operating at various levels: individual, social, environmental, institutional, and systemic. The model enables the systematic organization of these interrelationships, serving as a useful tool for planning multi-level interventions aimed at breastfeeding support [8]. At the individual level of the socio-ecological model, sociodemographic and interpersonal characteristics of the mother that shape breastfeeding behaviors are considered. This level comprises factors such as knowledge, attitudes towards breastfeeding, self-efficacy, and potentially competing demands, including professional employment and lifestyle. A substantial body of research has demonstrated that higher maternal educational attainment is associated with a greater prevalence of exclusive breastfeeding [9,10,11,12]. Attitudes towards breastfeeding are among the factors consistently associated with breastfeeding behaviors—more positive attitudes towards breastfeeding are related to a greater likelihood of its initiation and exclusive continuation [13,14]. Breastfeeding self-efficacy, defined as the mother’s belief in her own ability to successfully breastfeed, constitutes one of the key factors connected with initiation, duration, and exclusivity. Higher levels of this psychological resource are associated with a longer duration of breastfeeding and a greater probability of sustaining lactation, even in the presence of breastfeeding-related difficulties [15,16,17]. Furthermore, returning to professional work may constitute a barrier to continuing breastfeeding [18]. Socioeconomic status also plays a significant role, influencing both the initiation and continuation of breastfeeding, with a higher prevalence currently observed in developed countries among women of higher socioeconomic status [19].

At the interpersonal level, factors are primarily associated with receiving direct social support. Numerous studies have demonstrated that active paternal involvement in supporting breastfeeding significantly increases both its effectiveness and duration [20,21]. Furthermore, the family can function both as a supportive factor and as a barrier to breastfeeding [8]. Mothers, grandmothers, and mothers-in-law often play a significant role in promoting and supporting breastfeeding, encouraging women to initiate and continue lactation. Conversely, pressure from relatives and the transmission of negative or contradictory information regarding breastfeeding may hinder is exclusive maintenance and lead to earlier introduction of complementary feeding [18,22].

Obstetric and healthcare-related factors may also be associated with breastfeeding outcomes. Deliveries by cesarean section are frequently associated with delayed lactation initiation and breastfeeding-related [23]. Studies indicate that women who rate their childbirth experience positively exhibit higher levels of self-efficacy, which facilitates the initiation and maintenance of breastfeeding [24,25]. A positive childbirth experience, combined with strong relational resources, fosters a more favorable emotional climate for postpartum adaptation and the commencement of breastfeeding [13]. Hospital practices, such as early skin-to-skin contact, rooming-in, and support from healthcare staff can significantly influence effective lactation initiation and continuation [8,18].

At the policy and systemic level, regulatory frameworks pertaining to breastfeeding protection, the duration of maternity and parental leave, the conditions of a woman’s return to work, and workplace support are of considerable importance. Longer paid maternity leave and the possibility of expressing milk at work are conducive to maintaining exclusive breastfeeding [8,26,27]. Although broader organizational and policy factors may also be relevant, they were not directly measured in the present study.

Despite the growing recognition of the multifactorial nature of breastfeeding, limited research has simultaneously examined individual, interpersonal, and obstetric factors associated with exclusive breastfeeding in the Polish population within a single analytical framework. This study addresses this gap by utilizing the Socio-Ecological Model as a conceptual framework to systematically organize and interpret selected factors associated with exclusive breastfeeding.

Given the exploratory nature of this cross-sectional study and the limited prior research in the Polish population using the SEM-informed approach, we formulated the following general hypotheses:

1. Selected individual, interpersonal, and obstetric factors are significantly associated with exclusive breastfeeding;
2. Interpersonal factors, particularly partner support, exhibit the strongest association with exclusive breastfeeding.

The aim of this study was to identify individual, interpersonal, and obstetric factors associated with exclusive breastfeeding among Polish mothers, utilizing the Socio-Ecological Model as a conceptual framework to organize and interpret these relationships.

## 2. Materials and Methods

### 2.1. Study Design

The findings presented in this manuscript are derived from a larger research project titled “Health Status, Psychological and Sociodemographic Factors and Exclusive Breastfeeding in the Polish Female Population”. A cross-sectional design with an inclusive sampling approach was employed to investigate selected individual, interpersonal, and obstetric factors associated with exclusive breastfeeding among postpartum women in Poland. The study sample comprised 769 postpartum Polish mothers of infants and children up to 24 months of age who met the predefined inclusion criteria. Because ethnic minorities constitute a relatively small proportion of the Polish population, no stratification by ethnic background was performed. The study protocol was planned and described in accordance with STROBE guidelines for cross-sectional studies [28]. All research procedures were conducted in compliance with the ethical principles outlined in the 1964 World Medical Association (WMA) Declaration of Helsinki (with subsequent amendments) which govern research involving human participants. The study protocol was reviewed and approved by the Bioethics Committee of the Medical University of Gdańsk (decision No. KB/19/2025).

### 2.2. Study Setting

A cross-sectional survey design was employed to collect data via a self-administered online questionnaire. The study utilized the Computer-Assisted Web Interview (CAWI) method, which was selected to maximize geographical reach and facilitate participation among mothers of young children across Poland. Data collection was carried out between May and October 2025. A combination of convenience and snowball sampling strategies was applied, with recruitment conducted through publicly accessible online platforms and parenting-related social media groups operating nationwide. The research was conducted under the supervision of investigators from the Medical University of Gdańsk, ensuring standardized procedures throughout the study. To reduce the risk of selection bias, closed, regionally focused groups, and communities dedicated exclusively to breastfeeding, were deliberately excluded from the recruitment process. This approach was intended to capture a broad spectrum of infant feeding experiences and preferences. Nevertheless, the non-probabilistic nature of the sampling strategy limits the generalizability of the findings to the entire population of postpartum women in Poland.

### 2.3. Participants

Prior to study enrollment, all candidates were fully informed about the research objectives and methodology, and subsequently provided electronic informed consent.

Eligibility criteria included maternal age of 18 years or older, having an infant or toddler up to 24 months of age, and sufficient proficiency in Polish to independently complete the questionnaire. Mothers were eligible irrespective of their current or past infant feeding method (e.g., exclusive breastfeeding, mixed feeding, or formula feeding), allowing the study to capture a broad spectrum of experiences and practices. Exclusion criteria comprised maternal age below 18 years, insufficient proficiency in Polish, and multiple pregnancies.

Women with multiple pregnancies were excluded from the study. Multiple gestation was considered an exclusion criterion because it is associated with a distinct obstetric and neonatal clinical profile, including a higher frequency of preterm births, operative deliveries, low birth weight, and neonatal complications, which may independently affect breastfeeding initiation and continuation. This criterion was applied to reduce clinical heterogeneity within the study sample.

Recruitment was carried out via publicly accessible online platforms, and 769 mothers who met all eligibility criteria were included in the final analysis. The sample characteristics, including sociodemographic, perinatal, and psychometric variables, as well as their distribution according to the child’s age group, are presented in Table 1. Overall, 610 participants (79.3%) were classified as exclusively breastfeeding (EBF), whereas 159 (20.7%) were classified as non-EBF. At the time of survey completion, 82 participants (10.7%) had children aged 0–5 months, while 687 (89.3%) had children aged six months or older.

### 2.4. Data Collection Tools

Data were collected using a self-administered questionnaire developed by the authors in collaboration with a team of midwives, including two Certified Lactation Consultants. The instrument comprised closed-ended questions designed to capture key sociodemographic and perinatal characteristics, which were subsequently included in the analyses as control variables. These items addressed maternal age, educational level, self-rated financial status (assessed on a single-item, five-point Likert-type scale ranging from “very poor” to “very good”), parity (primiparous vs. multiparous), mode of delivery (vaginal or cesarean section), partner encouragement of breastfeeding, and the occurrence of complications during pregnancy. To ensure consistent interpretation, brief definitions of clinical terms were provided. The category “complications during pregnancy” encompassed both pre-existing chronic conditions and disorders that developed during its course, such as gestational hypertension, gestational diabetes and infections.

Infant feeding practices were assessed using the question: “How did you feed your child up to six months of age?”. The response categories were: (1) exclusive breastfeeding; (2) mixed feeding (breast milk and infant formula); (3) exclusive formula feeding; and (4) feeding exclusively with expressed breast milk.

Additionally, the postpartum psychosocial context was evaluated, including perceived social support, attitudes towards breastfeeding, and satisfaction with the childbirth experience. For this purpose, standardized psychometric instruments were employed. The authors of the Polish adaptations granted permission for their use.

The Multidimensional Scale of Perceived Social Support (MSPSS) was utilized in the Polish adaptation. The instrument comprises 12 items rated on a seven-point Likert scale, demonstrating high internal consistency in the Polish version (Cronbach’s *α* > 0.90) [29]. In the present study, the total MSPSS and its Significant Other, Family, and Friends subscales demonstrated excellent internal consistency (*α* = 0.938, 0.916, 0.915, and 0.953, respectively). Attitudes towards breastfeeding were measured using the Iowa Infant Feeding Attitudes Scale (IIFAS), adapted by Bień et al. for the Polish context. The scale consists of 17 statements rated on a five-point Likert scale (1–5), with nine items reverse-scored. A higher total score reflects a more positive attitude towards breastfeeding. The reliability of the Polish adaptation is satisfactory (Cronbach’s *α* = 0.725) [30]. In the present study, internal consistency for the IIFAS was good (*α* = 0.807).

Satisfaction with the childbirth experience was evaluated using the Birth Satisfaction Scale–Revised (BSS-R), a 10-item measure rated on a five-point Likert scale. For the purpose of this study, only the total score was utilized in the analyses. The Polish version of the scale demonstrates good psychometric properties, with Cronbach’s *α* values ranging from 0.79 to 0.87 [31,32]. In the present study, the internal consistency was good (*α* = 0.862).

This design of the research instrument enabled a multidimensional analysis of the factors associated with breastfeeding, encompassing individual, clinical, and psychosocial aspects, consistent with the assumptions of the Socio-Ecological Model.

The questionnaire was pilot-tested on a small independent sample of mothers (n = 10) to assess the clarity and comprehensibility of the questions, including the clinical terminology. Participants in the main study were also provided with a contact email address for any queries regarding the questionnaire.

### 2.5. Sample Size

An a priori sample size calculation was not performed; however, the absence of an a priori power calculation is acknowledged as a methodological limitation. In total, 1109 women initiated the survey, and the final analytical sample comprised 769 participants who met all eligibility criteria.

### 2.6. Bias

Several potential sources of bias should be considered when interpreting the findings of this study.

Selection bias may have been introduced due to the online, self-administered nature of the survey. Women with higher health literacy, a particular interest in lactation, or better internet access may have been more likely to participate, potentially underrepresenting mothers with limited digital skills or restricted online access.

Recall bias is another concern, as the study included mothers of children up to 24 months of age. Although this age limit was chosen to shorten the recall period, some participants may still have had difficulty accurately recalling details related to their childbirth, postpartum period, or breastfeeding practices. To mitigate this, respondents were explicitly instructed to answer all questions regarding social support and breastfeeding experiences with reference to the period when they were breastfeeding their youngest child. Nevertheless, the accuracy of retrospective self-reports may vary depending on the child’s age at the time of survey completion. The reports provided by mothers of older children may be subject to greater recall error due to the longer interval between the relevant childbirth, postpartum, and breastfeeding experiences, as well as survey completion.

The self-reported nature of the data also carries a risk of information bias, including social desirability bias—where respondents may provide answers perceived as socially acceptable—and declarative bias, which is associated with inaccurate or incomplete reporting. This is particularly relevant for questions concerning infant feeding practices and social support.

Although the questionnaire was administered in a fixed order to mitigate order effects, a potential priming effect cannot be excluded. Earlier questions regarding postpartum experiences and infant feeding may have influenced subsequent responses on the psychometric scales, particularly the IIFAS and the BSS-R. Additionally, the average completion time of approximately 20 min may have contributed to respondent fatigue, potentially reducing the precision of responses in the latter sections of the questionnaire.

To reduce selection bias, recruitment deliberately excluded private, local, and breastfeeding-focused online groups. However, this strategy may have inadvertently limited the representation of mothers with particularly strong breastfeeding commitments or those who actively seek lactation-related information.

Multiple pregnancies were excluded from the study to reduce clinical heterogeneity given their distinct obstetric and neonatal characteristics. However, this exclusion limits the generalizability of the findings to women with twin or higher-order pregnancies.

Missing data were addressed using multiple imputation by chained equations (MICE), and the robustness of the findings was assessed through a complete-case sensitivity analysis, which yielded results broadly consistent with the multiple-imputation analysis, supporting the stability of the primary associations observed.

Despite these potential biases, several strategies were implemented to enhance the methodological rigor of the study. These include using standardized psychometric instruments with validated properties in the Polish population, structuring questionnaire sections, and providing definitions for clinical terms.

However, these measures do not mitigate the inherent limitations of a cross-sectional design, retrospective self-report data, and non-probability online sampling.

### 2.7. Statistical Analysis

Data processing and statistical analyses were performed using IBM SPSS Statistics (Version 29). A hierarchical binary logistic regression analysis was employed to identify factors associated with exclusive breastfeeding. The dependent variable, “exclusive breastfeeding,” was defined as feeding the infant exclusively with breast milk—including expressed breast milk—during the first six months of life, without providing any other liquids or solid foods, except for vitamins, medications, or oral rehydration fluids, in accordance with the WHO definition [33]. The variable was constructed based on the question: “How did you feed your child during the first six months of life?” with the following response categories: (1) exclusive breastfeeding; (2) mixed feeding (breast milk and infant formula); (3) exclusive formula feeding; and (4) exclusive feeding with expressed breast milk. For the regression analysis, the outcome was dichotomized as 0 = non-EBF and 1 = EBF, with EBF specified as the modelled event. Because the exact duration of exclusive breastfeeding in months was not collected as a separate variable, the mean, median, or categorical distribution of EBF duration could not be determined. Furthermore, since 10.7% of participants had children aged 0–5 months at the time of survey completion, EBF classification in this subgroup could not represent the confirmed completion of the full six-month EBF period. Variables included in the regression models were organized into three hierarchical blocks informed by the SEM conceptual framework and encompassing selected sociodemographic, obstetric, attitudinal, and psychosocial factors. Categorical variables were coded as follows: education level (0 = below higher education and 1 = higher education); financial situation (0 = good and 1 = poor); parity (0 = multiparous and 1 = primiparous); mode of delivery (0 = cesarean delivery and 1 = vaginal delivery); complications/illnesses during pregnancy (0 = absent and 1 = present); and partner encouragement (0 = no and 1 = yes). The SEM was used to guide the conceptual organization of the variables rather than to represent a comprehensive operationalization of all levels of the model. Block 1 comprised maternal age, education level, financial situation, parity, mode of delivery, complications/illnesses during pregnancy, and child age. For the regression analysis, complications/illnesses during pregnancy were represented by a dichotomous composite variable (0 = no reported condition; 1 = at least one reported condition). This variable covered clinically heterogeneous conditions, including both pre-existing chronic diseases and conditions developing during pregnancy— it was not intended to represent a single specific obstetric complication. Block 2 additionally included breastfeeding attitudes measured using the Iowa Infant Feeding Attitude Scale. Finally, Block 3 involved birth satisfaction measured using the Birth Satisfaction Scale–Revised, the Family, Friends, and Significant Other subscales of the Multidimensional Scale of Perceived Social Support, and partner encouragement.

Linearity in the logit was assessed for all continuous predictors. A significant nonlinear association was identified for the IIFAS; therefore, it was centered around the sample mean (48.77), and a quadratic term (IIFAS^2^), calculated from this centered score, was included in the regression model. No significant departures from linearity in the logit were identified for the remaining continuous predictors.

Missing-data patterns were examined for all variables selected for the regression models. Missing values were present only for partner encouragement, with 45 missing observations (5.9% of the total sample; *N* = 769); no missing data were identified for the remaining variables included in the regression models. Missing data were handled using multiple imputation by chained equations (MICE), with 20 imputed datasets generated. The imputation model included all variables from the regression analyses and was specified according to their respective measurement levels. Logistic regression models were estimated separately in each imputed dataset, and regression coefficients, odds ratios (ORs), 95% confidence intervals (CIs), and *p*-values were pooled according to Rubin’s rules. Two sensitivity analyses were performed to assess the robustness of the findings. First, a complete-case analysis using the same model specification was conducted to evaluate the influence of missing-data handling. Second, the analysis was repeated after restricting the sample to mothers of children aged ≥6 months to evaluate the stability of the results with respect to the six-month EBF definition.

Model performance was evaluated using the *χ*^2^ model, Nagelkerke’s *R*^2^, and changes in *χ*^2^ (Δχ^2^) between successive models. Because these fit statistics are not directly pooled by the SPSS multiple-imputation procedure, values were reported as single estimates when identical across imputations or as ranges across the 20 imputed datasets when they varied between imputations. Calibration of the final model was assessed using the Hosmer–Lemeshow goodness-of-fit test, and discrimination was evaluated using the area under the receiver operating characteristic curve (AUROC). Multicollinearity was assessed using tolerance and variance inflation factor (VIF) statistics. Statistical significance was set at *p* < 0.05.

## 3. Results

### 3.1. Factors Associated with Exclusive Breastfeeding

Hierarchical logistic regression models were estimated using 20 multiply imputed datasets. Odds ratios (ORs), 95% confidence intervals (CIs), and *p*-values were pooled according to Rubin’s rules. Block 1 included age, education level, financial situation, parity, mode of delivery, complications/illnesses during pregnancy, and child age. IIFAS was entered in Block 2, while BSS-R, the MSPSS Family, Friends, and Significant Other subscales, and partner encouragement were entered in Block 3. The results are presented in Table 2.

The Hosmer–Lemeshow test was non-significant across the 20 multiply imputed datasets (*χ*^2^ = 4.01–10.03, *df* = 8, *p* = 0.263–0.856), indicating no evidence of poor model calibration. AUROC values ranged from 0.692 to 0.704, with corresponding 95% CIs between 0.646 and 0.750, indicating modest but stable discriminative performance across the imputed datasets. No significant departures from linearity in the logit were observed for age (*p* = 0.436), BSS-R (*p* = 0.925), MSPSS Friends (*p* = 0.684), MSPSS Family (*p* = 0.639), or MSPSS Significant Other (*p* = 0.914). In contrast, the IIFAS nonlinear term was significant (*p* = 0.003), supporting the quadratic specification used in the final model.

In Model 1, mode of delivery was significantly associated with EBF (OR = 1.67, 95% CI: 1.17–2.39, *p* = 0.005), as were complications or illnesses during pregnancy (OR = 1.62, 95% CI: 1.08–2.42, *p* = 0.020). Age, education level, financial situation, parity, and child age were not statistically significant. The χ^2^ model totalled 21.06, and Nagelkerke’s *R*^2^ was 0.042.

In Model 2, the addition of IIFAS increased the χ^2^ model to 42.54 (Δ*χ*^2^ = 21.48) and Nagelkerke’s *R*^2^ to 0.084. Both the linear IIFAS term (OR = 1.04, 95% CI: 1.00–1.09, *p* = 0.030) and the quadratic IIFAS term (OR = 0.99, 95% CI: 0.99–1.00, *p* = 0.001) were statistically significant.

In the final Model 3, mode of delivery (OR = 1.92, 95% CI: 1.32–2.80, *p* < 0.001), complications/illnesses during pregnancy (OR = 1.73, 95% CI: 1.14–2.64, *p* = 0.011), and partner encouragement (OR = 2.81, 95% CI: 1.72–4.59, *p* < 0.001) were significantly associated with EBF. The quadratic IIFAS term remained significant (OR = 0.99, 95% CI: 0.99–1.00, *p* = 0.002), whereas the linear IIFAS term was no longer statistically significant (OR = 1.04, 95% CI: 1.00–1.08, *p* = 0.078). BSS-R, MSPSS Family, MSPSS Friends, and MSPSS Significant Other were not independently associated with EBF in the final multivariable model. Maternal age, education level, financial situation, parity, and child age were also not independently associated with EBF.

### 3.2. Sensitivity Analyses

The complete-case analysis yielded results broadly consistent with the multiple-imputation analysis. Mode of delivery, complications/illnesses during pregnancy, partner encouragement, and the quadratic IIFAS term remained statistically significant, with comparable directions and sizes of the main associations. Detailed complete-case results are presented in Supplementary Table S1, and the direct comparison between the multiple-imputation and complete-case final models is provided in Supplementary Table S2. Among mothers of children aged ≥6 months, mode of delivery (OR = 2.18, 95% CI: 1.47–3.25, *p* < 0.001), complications/illnesses during pregnancy (OR = 1.67, 95% CI: 1.07–2.61, *p* = 0.025), and partner encouragement (OR = 2.66, 95% CI: 1.57–4.49, *p* < 0.001) remained statistically significant. The nonlinear association with IIFAS was also retained, demonstrating significant linear (OR = 1.05, 95% CI: 1.00–1.09, *p* = 0.037) and quadratic (OR = 0.99, 95% CI: 0.99–1.00, *p* = 0.008) terms. MSPSS Significant Other was not statistically significant (OR = 1.05, 95% CI: 0.99–1.11, *p* = 0.077), and the remaining predictors were not significant. Full results are presented in Supplementary Table S3.

### 3.3. Model Diagnostics

Across the 20 imputed datasets, Model 3 showed χ^2^ model values ranging from 62.92 to 70.00 and Nagelkerke’s *R*^2^ values between 0.123 and 0.136. The increase in χ^2^ associated with Block 3 ranged from 20.39 to 27.47. The correlations among the MSPSS subscales were moderate (r = 0.529–0.637), whereas correlations between partner encouragement and IIFAS, BSS-R, and the MSPSS subscales were weak (r = −0.012 to 0.045); the correlation matrix is provided in Supplementary Table S4. Tolerance values were within the range of 0.494 and 0.973, and VIF values, 1.028 and 2.025, indicating no problematic multicollinearity (Supplementary Table S5).

### 3.4. Summary Within the Socio-Ecological Framework

The SEM-informed analysis indicated that exclusive breastfeeding was associated with selected individual, interpersonal, and obstetric factors included in the present study. Among the interpersonal variables examined, direct partner encouragement showed the largest observed association with EBF. However, given the cross-sectional design and the use of a single binary item to assess partner encouragement, this finding should be interpreted cautiously and does not establish that partner encouragement has greater causal influence than broader perceived social support. At the individual level, breastfeeding attitudes showed a nonlinear association with exclusive breastfeeding: the predicted probability of EBF increased with IIFAS scores up to approximately 51 points, and then subsequently decreased. This suggests that the observation between breastfeeding attitudes and exclusive breastfeeding may be nonlinear rather than adequately represented by a simple linear relationship. Obstetric characteristics were also associated with EBF. Vaginal delivery was related to higher odds of exclusive breastfeeding compared with cesarean delivery. The presence of complications/illnesses during pregnancy was also associated with higher odds of EBF. Given the heterogeneity of this composite variable and the unexpected direction of the association, this finding should be interpreted cautiously.

In contrast, broader perceived social support from family, friends, and significant others was not independently associated with EBF after adjustment for the other variables. These findings indicate that partner encouragement and the MSPSS subscales showed different patterns of association with EBF in the present model; however, direct comparisons regarding the relative importance of specific versus general support should be made with caution.

**Figure 1:**
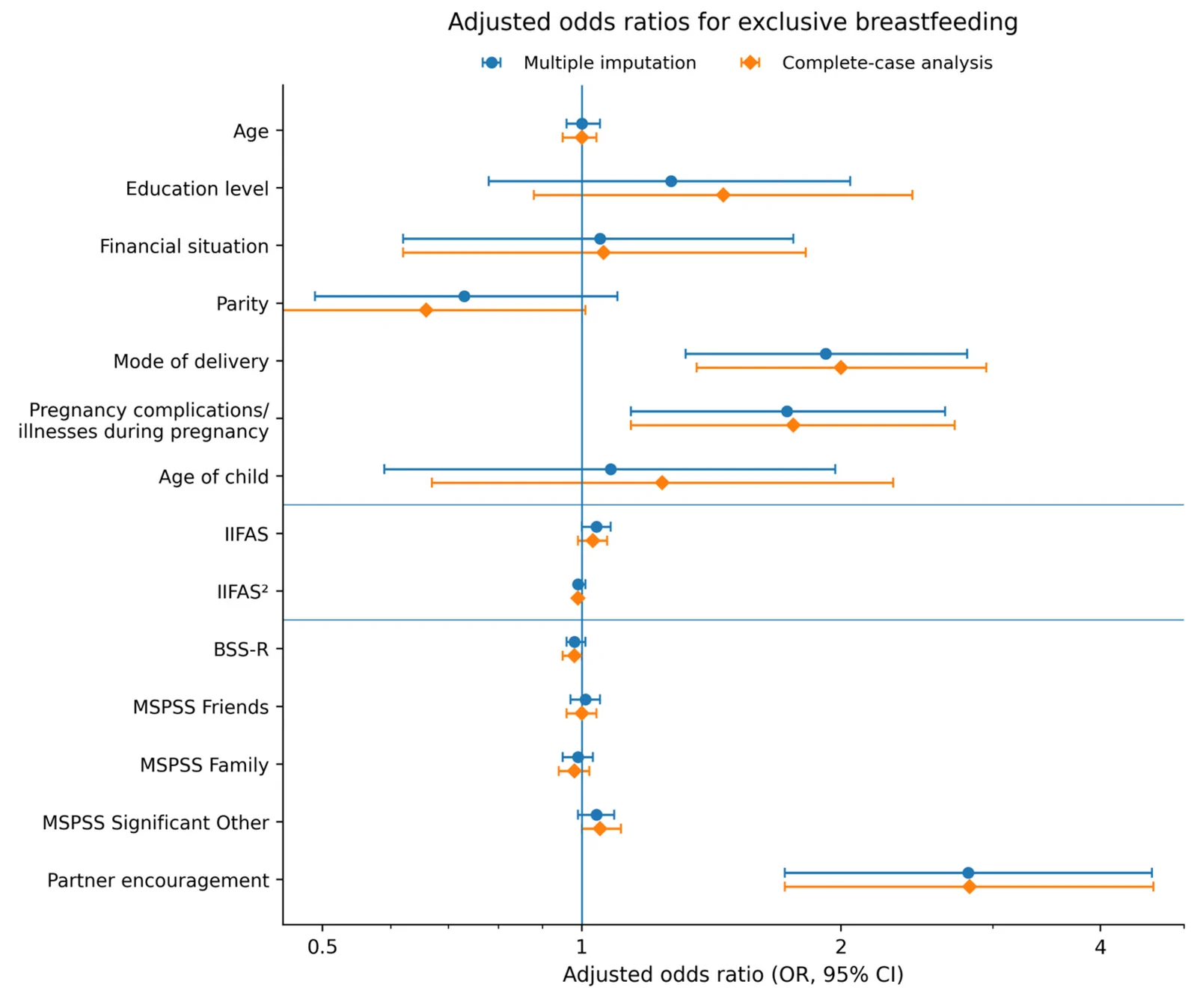
Adjusted odds ratios for exclusive breastfeeding: multiple-imputation and complete-case analyses. Note. Event modelled: exclusive breastfeeding (EBF) = 1; non-EBF = 0. Binary-variable comparisons: higher vs. below higher education; poor vs. good financial situation; primiparous vs. multiparous; vaginal vs. cesarean delivery; complications/illnesses during pregnancy present vs. absent; and partner encouragement yes vs. no.

Taken together, the findings show that EBF was associated with selected individual, interpersonal, and obstetric factors included in the present analysis. Partner encouragement exhibited the largest observed association among the interpersonal variables examined, while breastfeeding attitudes demonstrated a nonlinear association with EBF. Maternal age, education, financial situation, parity, child age, and the MSPSS subscales were not independently associated with EBF in the final model. These findings should be interpreted within the limited set of SEM-informed variables assessed in the present study. Figure 1 presents the adjusted odds ratios from the multiple-imputation and complete-case analyses.

## 4. Discussion

The present study examined selected factors associated with exclusive breastfeeding within an SEM-informed framework. Among the variables included in the final model, partner encouragement showed the largest observed association with EBF.

It should be noted that the proportion of participants classified as practicing EBF in the present study should not be interpreted as a population estimate of adherence to the WHO recommendation of six completed months of exclusive breastfeeding. At the time of survey completion, 10.7% of participants had children aged 0–5 months and had therefore not yet completed the full six-month period. Accordingly, the reported EBF proportion reflects the outcome classification applied in the present study and should be interpreted in the context of child-age distribution.

The SEM was used as a conceptual framework to organize and interpret selected individual, interpersonal, and obstetric factors. Community, sociocultural, and policy-level influences were not directly measured. Thus, the findings should be interpreted as being informed by selected domains of the SEM rather than as representing a comprehensive operationalization of the model.

### 4.1. Interpersonal Factors: Partner Support

Among the variables included in the final model, partner encouragement showed the largest observed association with exclusive breastfeeding, women reporting partner encouragement having higher odds of EBF. This finding is consistent with the relevance of interpersonal support within the SEM framework, in which relationships within the mother’s immediate environment are considered potentially important for health behaviors. Although partner encouragement and perceived social support measured by the MSPSS are conceptually related, they represent distinct aspects of support. The MSPSS captures broader perceived support from family, friends, and significant others, whereas partner encouragement reflects a specific breastfeeding-related behavior of the partner. Their simultaneous inclusion in the final model was therefore intended to distinguish general perceived social support from breastfeeding-specific partner encouragement.

These findings are consistent with previous research highlighting partner support as one of the key factors related to the initiation and continuation of breastfeeding [20,34,35]. Partner involvement and encouragement have been associated with greater maternal confidence and breastfeeding self-efficacy, which, in turn, are associated with improved breastfeeding outcomes. A study by Mannion et al. demonstrated that the presence of the father during breastfeeding, as well as active assistance—such as physically preparing the infant for feeding or sharing household responsibilities during the early postpartum period—was perceived as positive support associated with breastfeeding decisions.

However, given the cross-sectional design and the use of a single binary item to assess partner encouragement, the observed association should not be interpreted as causal. Within the SEM framework, these findings suggest that future prospective and intervention studies should further examine the role of partner involvement and breastfeeding-specific support alongside individual-level factors.

### 4.2. Obstetric and Clinical Factors: Mode of Delivery and Complications During Pregnancy

Mode of delivery, complications during pregnancy, and birth satisfaction were among the obstetric and clinical factors examined in the present study. Although these factors may be influenced by the healthcare context, they should not be interpreted as direct measures of the institutional level of the SEM. Our study indicates that women who delivered vaginally had nearly twice the odds of exclusive breastfeeding compared with those who delivered by cesarean section. The observed association between mode of delivery and EBF is consistent with previous studies reporting that cesarean delivery may be associated with delayed breastfeeding initiation and greater early breastfeeding difficulties [36,37] Previous research suggests that differences in early postpartum care, including opportunities for immediate skin-to-skin contact and mother–infant separation, may partly contribute to breastfeeding differences following cesarean and vaginal delivery [38]. However, these aspects of perinatal care were not directly assessed in the present study and should therefore be considered possible explanations rather than mechanisms demonstrated by our data.

Previous studies have also associated supportive hospital practices, including those promoted by the Baby-Friendly Hospital Initiative, with improved breastfeeding outcomes [39,40]. However, hospital-level breastfeeding practices were not directly assessed in the present study.

#### Complications During Pregnancy—An Unexpected Association

An important finding of this study was that the presence of complications during pregnancy were associated with higher odds of exclusive breastfeeding. This result should be interpreted cautiously because the composite pregnancy-complications variable encompassed heterogeneous clinical conditions with potentially different relationships with breastfeeding. This result appears counterintuitive in light of previous research, indicating that complications during pregnancy or delivery may hinder lactation initiation and maintenance [25,41,42,43], and given that findings from our earlier studies suggest that pregnancy complications were not significantly associated with breastfeeding attitudes [44]. In the present study, the analysis of complications referred to the occurrence of conditions before or during pregnancy, encompassing both pre-existing chronic diseases and disorders that developed during gestation (e.g., gestational hypertension, gestational diabetes, infections).

Given the unexpected direction of this association, several factors may account for this finding, including exposure heterogeneity, differences in healthcare utilization, residual confounding, variations in condition severity, or chance. One possible explanation suggested by previous literature is that women experiencing complications during pregnancy may have more frequent contact with healthcare services, potentially increasing opportunities for professional lactation support and education [45]. However, healthcare-contact intensity and professional lactation support were not directly assessed in the present study; therefore, this interpretation remains hypothetical. Consequently, the observed association should be regarded as exploratory and not as evidence of a causal relationship between pregnancy complications and EBF. Further studies employing clinically more homogeneous categories of complications during pregnancy are warranted to clarify this finding.

### 4.3. Individual Factors: Breastfeeding Attitudes and Maternal Background Characteristics

At the individual level, breastfeeding attitudes showed a nonlinear association with exclusive breastfeeding. The predicted probability of EBF increased with IIFAS scores up to approximately 51 points, after which it subsequently decreased, indicating an inverted U-shaped pattern. This finding suggests that the relationship between breastfeeding attitudes and EBF may be more complex than a simple linear association. One possible explanation is that the IIFAS assesses attitudes towards infant feeding rather than the actual capacity to maintain exclusive breastfeeding. Very high IIFAS scores may reflect particularly strong or idealized beliefs regarding breastfeeding, which may not necessarily translate into practice when practical, physiological, or contextual difficulties arise. However, this interpretation remains speculative, and the observed nonlinear association requires confirmation in future studies.

Previous research indicates that attitudes constitute important individual-level factors associated with health behaviors, including both intentions and actual maternal practices. This finding aligns with the assumptions of the SEM, according to which attitudes represent an individual-level factor that is shaped by influences operating at higher levels—interpersonal, institutional, and sociocultural. Previous studies have similarly reported associations between more favorable breastfeeding attitudes and breastfeeding behaviors, although attitudes towards infant feeding may be multidimensional and context-dependent [46,47,48,49].

In the final logistic regression model, background characteristics—including maternal age, education, financial situation, and parity—were not significantly associated with EBF after adjustment for the other variables included in the model. These variables were also not statistically significant in the initial model containing sociodemographic and obstetric factors, suggesting that no independent associations with EBF were identified within the present analysis.

### 4.4. Birth Satisfaction and Exclusive Breastfeeding

In the present study, birth satisfaction—measured via the BSS-R—was not significantly associated with exclusive breastfeeding after adjustment for the other variables included in the final model.

Birth satisfaction reflects a woman’s subjective evaluation of her childbirth experience, and may be influenced by both individual perceptions and the context of perinatal care. Nevertheless, previous research suggests that childbirth experience may be relevant to postpartum psychosocial outcomes. Studies have reported that a positive childbirth experience may be associated with better postpartum adaptation, higher breastfeeding self-efficacy, and more favorable attitudes towards breastfeeding [50].

However, the present study did not examine mediation or other indirect pathways; therefore, no conclusions regarding such mechanisms can be drawn from these data. Further longitudinal research, specifically designed to evaluate the temporal and potentially indirect relationships between childbirth experience and breastfeeding outcomes, is warranted.

### 4.5. Strengths and Limitations

This study has several notable strengths. It employed Polish adaptations of validated psychometric instruments, which ensured measurement reliability and facilitated cross-cultural comparisons with other studies. The questionnaire covered a broad range of sociodemographic, obstetric, and psychosocial variables, enabling a multidimensional assessment of factors associated with exclusive breastfeeding.

Furthermore, utilizing the SEM as a conceptual framework enabled the organization and interpretation of selected individual, interpersonal, and obstetric factors within a broader multilevel perspective.

However, several limitations should be acknowledged. Primarily, the sample size was determined pragmatically without a priori power analysis, which represents a methodological constraint. Additionally, the cross-sectional design precludes causal inferences. The use of a self-administered online questionnaire, while enabling efficient nationwide recruitment and reducing logistical burden, limited the opportunity to clarify responses and may have introduced selection bias. Another limitation is the overrepresentation of women with higher education in the study sample compared to national statistics for reproductive-aged women in Poland [51]. This pattern is common in studies using open online recruitment, which tend to attract individuals with greater familiarity with digital technologies, higher health awareness and more proactive engagement in parenting communities. Consequently, the use of convenience sampling and online recruitment may limit the external validity of the findings, as women with higher digital literacy, greater interest in breastfeeding or better access to online platforms were more likely to participate. Furthermore, participants’ geographical location was not collected; thus, the regional distribution of the sample could not be assessed, and geographical representativeness across Poland cannot be established. Therefore, the results should be interpreted with caution and cannot be directly generalized to the entire population of Polish mothers, particularly those with lower educational attainment or limited Internet access. The use of retrospective self-report carries a risk of recall bias and social desirability effects.

Partner encouragement was assessed using a single binary item, which did not capture the multidimensional nature of partner support, including emotional, instrumental, informational, or potentially negative forms of involvement. This measurement approach may have limited the precision with which partner support was evaluated, and may also have been susceptible to social-desirability bias. As a result, the observed association between partner encouragement and EBF should be interpreted with caution. Future studies should employ validated multi-item scales specifically designed to assess different dimensions of partner support for breastfeeding.

Missing data were addressed using multiple imputation by chained equations, with estimates pooled across 20 imputed datasets according to Rubin’s rules. In addition, a complete-case sensitivity analysis yielded results broadly consistent with the multiple-imputation analysis, supporting the robustness of the primary associations observed.

An additional limitation concerns the variation in child age at the time of survey completion. In 10.7% of the participants, the child was aged 0–5 months and had therefore not yet completed the full six-month period. Consequently, EBF classification in this subgroup cannot be interpreted as confirmed completion of six-month EBF. Conversely, mothers of older children reported past feeding practices retrospectively, which may have increased the risk of recall bias as the time elapsed since birth increased. These differences in the EBF evaluation window and in the duration of retrospective recall may have introduced age-dependent misclassification. Accordingly, the proportion of EBF observed in the present study should not be interpreted as a population estimate of adherence to the WHO’s recommendation of six completed months of exclusive breastfeeding. Importantly, a sensitivity analysis restricted to mothers of children aged six months or older yielded findings broadly consistent with the primary analysis. Mode of delivery, gestational complications/illnesses, partner encouragement, and the nonlinear association with IIFAS remained statistically significant, supporting the robustness of the primary findings when the analysis was restricted to participants whose children had reached at least six months of age.

Several potentially important factors associated with breastfeeding were not comprehensively assessed in the present study, including infant sex, gestational age and preterm birth, birth weight, neonatal intensive-care admission, early skin-to-skin contact and breastfeeding initiation, rooming-in, breastfeeding difficulties and perceived milk insufficiency, maternal pre-pregnancy BMI (Body Mass Index), smoking, employment and return-to-work status, maternity-leave use, workplace breastfeeding support, postpartum contraception, return of menstruation and lactational amenorrhea, previous breastfeeding experience, professional lactation support, and postpartum depression or anxiety. The omission of these variables may have resulted in residual confounding and partly account for the limited explanatory power of the final model. In particular, previous breastfeeding experience was not assessed, and parity alone cannot distinguish mothers with successful previous breastfeeding experience from those without one. Consequently, the observed associations should be interpreted cautiously and not be considered evidence of causal relationships. In addition, the composite pregnancy-complications variable combined clinically heterogeneous conditions with potentially different severity, management, and implications for breastfeeding. This heterogeneity may have obscured condition-specific associations and may have contributed to the unexpected direction of the observed association. Therefore, the finding regarding pregnancy complications should be considered exploratory and interpreted with caution.

Despite the statistically significant associations identified in the final model, its overall explanatory capacity remained modest, with Nagelkerke’s *R*^2^ values ranging from 0.123 to 0.136 across the 20 imputed datasets. This indicates that a substantial proportion of the variation in exclusive breastfeeding remained unexplained. Although pseudo-*R*^2^ values should not be interpreted equivalently to *R*^2^ in linear regression, these findings suggest that other unmeasured clinical, physiological, sociocultural, community, and policy-related factors may contribute to breastfeeding outcomes. Therefore, the observed associations should be interpreted cautiously, and future studies should incorporate a broader range of relevant factors.

Despite these limitations, this study contributes to a deeper understanding of factors associated with exclusive breastfeeding across multiple levels of the Socio-Ecological Model. The utilization of standardized instruments and an SEM-informed analytical framework provides a foundation for future longitudinal and mixed-methods research integrating psychosocial, medical, biological, sociocultural, community, and policy-related factors.

## 5. Conclusions

The findings of this study align with the general assumptions of the Socio-Ecological Model and suggest that exclusive breastfeeding is associated with factors operating across multiple domains. However, the present study examined only selected individual, interpersonal, and obstetric factors and did not directly assess community, sociocultural, or policy-level influences. Future research should incorporate these broader levels to provide a more comprehensive evaluation of the factors associated with exclusive breastfeeding. Among the variables included in the final model, partner encouragement showed the strongest association with EBF. This finding is consistent with the relevance of immediate social environment to breastfeeding behavior and highlights partner encouragement as an important interpersonal factor warranting further investigation. Obstetric factors related to pregnancy and delivery—specifically mode of delivery and complications/illnesses during pregnancy—were also significantly associated with exclusive breastfeeding.

At the individual level, breastfeeding attitudes showed a nonlinear association with exclusive breastfeeding. The predicted probability of EBF increased alongside IIFAS scores up to approximately 51 points, and then subsequently decreased. This finding indicates that the relationship between breastfeeding attitudes and EBF may be more complex than a simple linear association and requires confirmation in future studies.

Selected individual-level characteristics, including maternal age, education, financial situation, and parity, were not significantly connected with EBF after adjustment for the other variables included in the final model.

From a practical standpoint, these findings indicate that breastfeeding support may benefit from considering not only individual-level factors but also the broader social and healthcare context. In particular, the observed association between partner encouragement and EBF may inform future prospective and intervention studies evaluating partner-focused lactation support. Similarly, the associations noted for obstetric factors highlight the need for further research into the role of perinatal care in breastfeeding outcomes. Given the cross-sectional design of the present study, these results should not be interpreted as evidence that modifying these factors would increase exclusive breastfeeding rates.

## Figures and Tables

**Table 1:** Characteristics of the study sample by child age group.

Variable		Whole Group (*N* = 769)		Child Age Group			
				0–5 Months (*N* = 82; 10.7%)		≥6 Months (*N* = 687; 89.3%)	
Age		32.24	4.85	31.30	4.52	32.35	4.88
MSPSS—Multidimensional Scale of Perceived Social Support		64.48	14.48	65.74	8.25	64.33	15.04
Significant Other		22.89	5.05	23.06	3.18	22.87	5.23
Family		20.82	5.94	20.40	5.14	20.87	6.03
Friends		20.76	6.11	21.40	4.60	20.69	6.27
IIFAS—Iowa Infant Feeding Attitude Scale		48.77	4.44	48.23	4.15	48.84	4.47
BSS-R—Birth Satisfaction Scale–Revised		23.08	7.78	23.26	7.75	23.06	7.79
Education level	Primary	2	0.3	0	0.0	2	0.3
	Vocational	9	1.2	1	1.2	8	1.2
	Secondary	121	15.7	21	25.6	100	14.6
	Higher (Bachelor’s/Master’s degree)	637	82.8	60	73.2	577	84.0
Financial situation	Very good	206	26.8	23	28.0	183	26.6
	Good	450	58.5	49	59.8	401	58.4
	Satisfactory	110	14.3	10	12.2	100	14.6
	Bad	2	0.3	0	0.0	2	0.3
	Very bad	1	0.1	0	0.0	1	0.1
Parity	Multiparous	298	38.8	45	54.9	253	36.8
	Primiparous	471	61.2	37	45.1	434	63.2
Mode of delivery	Caesarean section	297	38.6	32	39.0	265	38.6
	Vaginal delivery	472	61.4	50	61.0	422	61.4
Partner encouragement of breastfeeding	No	97	13.4	10	13.2	87	13.4
	Yes	627	86.6	66	86.8	561	86.6
Pregnancy complications/illnesses during pregnancy	No	515	67.0	52	63.4	463	67.4
	Yes	254	33.0	30	36.6	224	32.6
EBF	No	159	20.7	17	20.7	142	20.7
	Yes	610	79.3	65	79.3	545	79.3

**Table 2:** Hierarchical logistic regression models of factors associated with exclusive breastfeeding: multiple-imputation analysis.

Predictor	Model 1 OR (95% CI)	*p*	Model 2 OR (95% CI)	*p*	Model 3 OR (95% CI)	*p*
Block 1						
Age	0.99 (0.96–1.03)	0.792	0.99 (0.95–1.03)	0.545	1.00 (0.96–1.05)	0.894
Education level	1.46 (0.92–2.30)	0.105	1.46 (0.92–2.31)	0.110	1.27 (0.78–2.05)	0.333
Financial situation	1.08 (0.66–1.77)	0.749	1.15 (0.70–1.89)	0.586	1.05 (0.62–1.76)	0.860
Parity	0.71 (0.48–1.05)	0.088	0.71 (0.48–1.07)	0.102	0.73 (0.49–1.10)	0.137
Mode of delivery	1.67 (1.17–2.39)	0.005	1.80 (1.25–2.60)	0.002	1.92 (1.32–2.80)	<0.001
Complications/illnesses during pregnancy	1.62 (1.08–2.42)	0.020	1.69 (1.12–2.56)	0.013	1.73 (1.14–2.64)	0.011
Age of child	1.04 (0.58–1.87)	0.886	1.05 (0.58–1.89)	0.880	1.08 (0.59–1.97)	0.813
Block 2						
IIFAS (centered)	—	—	1.04 (1.00–1.09)	0.030	1.04 (1.00–1.08)	0.078
IIFAS^2^ (centered)	—	—	0.99 (0.99–1.00)	0.001	0.99 (0.99–1.00)	0.002
Block 3						
BSS-R	—	—	—	—	0.98 (0.96–1.01)	0.125
MSPSS Friends	—	—	—	—	1.01 (0.97–1.05)	0.767
MSPSS Family	—	—	—	—	0.99 (0.95–1.03)	0.618
MSPSS Significant Other	—	—	—	—	1.04 (0.99–1.09)	0.133
Partner encouragement	—	—	—	—	2.81 (1.72–4.59)	<0.001
Model fit						
Model χ^2^ (*df*)	21.06	—	42.54	—	62.92–70.00	—
Nagelkerke’s *R*^2^	0.042	—	0.084	—	0.123–0.136	—
Δχ^2^ (*df*)	—	—	21.48	—	20.39–27.47	—

Note. Event modelled: exclusive breastfeeding (EBF) = 1; non-EBF = 0. Binary-variable comparisons were coded as follows: higher vs. below higher education (1 vs. 0); poor vs. good financial situation (1 vs. 0); primiparous vs. multiparous (1 vs. 0); vaginal vs. cesarean delivery (1 vs. 0); complications/illnesses during pregnancy present vs. absent (1 vs. 0); and partner encouragement yes vs. no (1 vs. 0). IIFAS was centered around its sample mean (48.77); the quadratic term (IIFAS^2^) was calculated from the centered IIFAS score and was included to assess nonlinearity in the association between IIFAS and exclusive breastfeeding. ORs, 95% CIs, and *p*-values are pooled estimates from 20 multiple imputed datasets according to Rubin’s rules.

## Data Availability

The original contributions presented in the study are included in the article; further inquiries can be directed to the corresponding author.

## Supplementary Materials

The following supporting information can be downloaded at: https://www.mdpi.com/article/10.3390/nu18172900/s1, Table S1: Hierarchical logistic regression models of factors associated with exclusive breastfeeding: complete-case sensitivity analysis. Table S2: Comparison of the final logistic regression model based on multiple imputation and complete-case analysis. Table S3: Comparison of the final logistic regression model between the full sample and mothers of children aged ≥6 months. Table S4: Correlations among IIFAS, BSS-R, MSPSS subscales, and partner encouragement. Table S5: Multicollinearity diagnostics for predictors included in the final model.

## Abbreviations

The following abbreviations are used in this manuscript:

BSS-R	Birth Satisfaction Scale–Revised
CAWI	Computer-Assisted Web Interview
IIFAS	Iowa Infant Feeding Attitudes Scale
MSPSS	Multidimensional Scale of Perceived Social Support
SEM	Socio-Ecological Model
STROBE	Strengthening the Reporting of Observational Studies in Epidemiology
UNICEF	United Nations Children’s Fund
WHO	World Health Organization
WMA	World Medical Association
