# Supplementary material for: Individual, Interpersonal, and Obstetric Factors Associated with Exclusive Breastfeeding Among Polish Mothers: A Cross-Sectional Study Informed by the Socio-Ecological Model
Source: Nutrients. 2026 Sep 4;18(17):2900. doi: 10.3390/nu18172900 (PMC13567125; doi:10.3390/nu18172900)
Supplement: Supplementary file 1 [file nutrients-18-02900-s001.zip › Table S1.pdf]

Supplementary Table S1. Hierarchical logistic regression models of factors associated with exclusive breastfeeding: complete-case sensitivity analysis

Factor	Model 1 OR (95% CI)	*p*	Model 2 OR (95% CI)	*p*	Model 3 OR (95% CI)	*p*
Block 1						
Age	0.99 (0.95–1.03)	0.551	0.98 (0.94–1.02)	0.377	1.00 (0.95–1.04)	0.849
Education level	1.68 (1.04–2.73)	0.035	1.67 (1.02–2.72)	0.040	1.46 (0.88–2.42)	0.146
Financial situation	1.10 (0.65–1.83)	0.729	1.17 (0.69–1.97)	0.557	1.06 (0.62–1.82)	0.832
Parity	0.64 (0.42–0.96)	0.033	0.64 (0.42–0.98)	0.038	0.66 (0.43–1.01)	0.057
Mode of delivery	1.73 (1.20–2.51)	0.004	1.87 (1.28–2.73)	0.001	2.00 (1.36–2.95)	<0.001
Complications/illnesses during pregnancy	1.63 (1.08–2.48)	0.021	1.70 (1.11–2.61)	0.014	1.76 (1.14–2.71)	0.011
Age of child	1.20 (0.66–2.17)	0.557	1.20 (0.65–2.19)	0.561	1.24 (0.67–2.30)	0.499
Block 2						
IIFAS (centered)	—	—	1.04 (1.00–1.08)	0.063	1.03 (0.99–1.07)	0.159
$IIFAS^2$ (centered)	—	—	0.99 (0.99–1.00)	0.001	0.99 (0.99–1.00)	0.002
Block 3						
BSS-R	—	—	—	—	0.98 (0.95–1.00)	0.098
MSPSS Friends	—	—	—	—	1.00 (0.96–1.04)	0.847
MSPSS Family	—	—	—	—	0.98 (0.94–1.02)	0.361
MSPSS Significant Other	—	—	—	—	1.05 (1.00–1.11)	0.076
Partner encouragement	—	—	—	—	2.82 (1.72–4.61)	<0.001
Model fit						
Model χ^2 (df)	24.25 (7)		43.68 (9)		66.89 (14)	
Nagelkerke's R^2	0.052		0.092		0.138	
$\Delta\chi^2$ (df)	—		19.43 (2)		23.21 (5)	

Note. IIFAS was centered around its sample mean (48.77); the quadratic term ($IIFAS^2$) was calculated from the centered IIFAS score and included to assess nonlinearity in the association between IIFAS and exclusive breastfeeding. Complete-case analysis included 724 participants with complete data on all variables included in the final model.
