# Supplementary material for: Individual, Interpersonal, and Obstetric Factors Associated with Exclusive Breastfeeding Among Polish Mothers: A Cross-Sectional Study Informed by the Socio-Ecological Model
Source: Nutrients. 2026 Sep 4;18(17):2900. doi: 10.3390/nu18172900 (PMC13567125; doi:10.3390/nu18172900)
Supplement: Supplementary file 1 [file nutrients-18-02900-s001.zip › Table S2.pdf]

Supplementary Table S2. Comparison of the final logistic regression model based on multiple imputation and complete-case analysis

Predictor	Multiple imputation OR (95% CI)	*p*	Complete-case OR (95% CI)	*p*
Age	1.00 (0.96–1.05)	0.894	1.00 (0.95–1.04)	0.849
Education level	1.27 (0.78–2.05)	0.333	1.46 (0.88–2.42)	0.145
Financial situation	1.05 (0.62–1.76)	0.860	1.06 (0.62–1.82)	0.832
Parity	0.73 (0.49–1.10)	0.137	0.66 (0.43–1.01)	0.057
Mode of delivery	1.92 (1.32–2.80)	<0.001	2.00 (1.36–2.95)	<0.001
Pregnancy complications/illnesses	1.73 (1.14–2.64)	0.011	1.76 (1.14–2.71)	0.011
Age of child	1.08 (0.59–1.97)	0.813	1.24 (0.67–2.30)	0.499
IIFAS (centered)	1.04 (1.00–1.08)	0.078	1.03 (0.99–1.07)	0.159
$IIFAS^2$ (centered)	0.99 (0.99–1.00)	0.002	0.99 (0.99–1.00)	0.002
BSS-R	0.98 (0.96–1.01)	0.125	0.98 (0.95–1.00)	0.098
MSPSS Friends	1.01 (0.97–1.05)	0.767	1.00 (0.96–1.04)	0.847
MSPSS Family	0.99 (0.95–1.03)	0.618	0.98 (0.94–1.02)	0.361
MSPSS Significant Other	1.04 (0.99–1.09)	0.133	1.05 (1.00–1.11)	0.076
Partner encouragement	2.81 (1.72–4.59)	<0.001	2.82 (1.72–4.61)	<0.001
