# Supplementary material for: Individual, Interpersonal, and Obstetric Factors Associated with Exclusive Breastfeeding Among Polish Mothers: A Cross-Sectional Study Informed by the Socio-Ecological Model
Source: Nutrients. 2026 Sep 4;18(17):2900. doi: 10.3390/nu18172900 (PMC13567125; doi:10.3390/nu18172900)
Supplement: Supplementary file 1 [file nutrients-18-02900-s001.zip › Table S3.pdf]

Supplementary Table S3. Comparison of the final logistic regression model between the full sample and mothers of children aged ≥6 months

Predictor	Multiple imputation OR (95% CI)	*p*	Children ≥6 months: MICE OR (95% CI)	*p*
Age	1.00 (0.96–1.05)	0.894	1.01 (0.96–1.05)	0.756
Education level	1.27 (0.78–2.05)	0.333	1.33 (0.79–2.23)	0.280
Financial situation	1.05 (0.62–1.76)	0.860	1.07 (0.62–1.85)	0.796
Parity	0.73 (0.49–1.10)	0.137	0.76 (0.49–1.18)	0.215
Mode of delivery	1.92 (1.32–2.80)	<0.001	2.18 (1.47–3.25)	<0.001
Complications/illnesses during pregnancy	1.73 (1.14–2.64)	0.011	1.67 (1.07–2.61)	0.025
IIFAS (centered)	1.08 (0.59–1.97)	0.813	1.05 (1.00–1.09)	0.037
$IIFAS^2$ (centered)	1.04 (1.00–1.08)	0.078	0.99 (0.99–1.00)	0.008
BSS-R	0.99 (0.99–1.00)	0.002	0.98 (0.96–1.01)	0.151
MSPSS Friends	0.98 (0.96–1.01)	0.125	0.99 (0.96–1.04)	0.804
MSPSS Family	1.01 (0.97–1.05)	0.767	0.99 (0.94–1.03)	0.573
MSPSS Significant Other	0.99 (0.95–1.03)	0.618	1.05 (0.99–1.11)	0.077
Partner encouragement	1.04 (0.99–1.09)	0.133	2.66 (1.57–4.49)	<0.001
