# Supplementary material for: Individual, Interpersonal, and Obstetric Factors Associated with Exclusive Breastfeeding Among Polish Mothers: A Cross-Sectional Study Informed by the Socio-Ecological Model
Source: Nutrients. 2026 Sep 4;18(17):2900. doi: 10.3390/nu18172900 (PMC13567125; doi:10.3390/nu18172900)
Supplement: Supplementary file 1 [file nutrients-18-02900-s001.zip › Table S4.pdf]

Supplementary Table S4. Correlations among IIFAS, BSS-R, MSPSS subscales, and partner encouragement

Variable	Partner encouragement	IIFAS	BSS-R	MSPSS Friends	MSPSS Family	MSPSS Significant Other
Partner encouragement	1.000					
IIFAS	0.031	1.000				
BSS-R	−0.002	−0.125	1.000			
MSPSS Friends	−0.012	−0.019	0.097**	1.000		
MSPSS Family	0.010	0.002	0.075*	0.529	1.000	
MSPSS Significant Other	0.045	0.003	0.170	0.565	0.637	1.000

Note. Correlations are based on the combined results across the 20 multiply imputed datasets. For the binary partner-encouragement variable, Pearson's correlation with continuous variables is equivalent to the point-biserial correlation. *$p < 0.05$; **$p < 0.01$; ***$p < 0.001$.
