# Supplementary material for: Individual, Interpersonal, and Obstetric Factors Associated with Exclusive Breastfeeding Among Polish Mothers: A Cross-Sectional Study Informed by the Socio-Ecological Model
Source: Nutrients. 2026 Sep 4;18(17):2900. doi: 10.3390/nu18172900 (PMC13567125; doi:10.3390/nu18172900)
Supplement: Supplementary file 1 [file nutrients-18-02900-s001.zip › Table S5.pdf]

Supplementary Table S5. Multicollinearity diagnostics for predictors included in the final model

Predictor	Tolerance	VIF
Age	0.804–0.818	1.222–1.243
Education level	0.915–0.937	1.067–1.093
Financial situation	0.965–0.973	1.028–1.035
Parity	0.862–0.868	1.152–1.160
Mode of delivery	0.964–0.965	1.036–1.038
Complications/illnesses during pregnancy	0.968–0.971	1.030–1.033
Age of child	0.960	1.041–1.042
IIFAS (centered)	0.915–0.923	1.083–1.093
$IIFAS^2$ (centered)	0.915–0.923	1.083–1.093
BSS-R	0.934–0.942	1.062–1.070
MSPSS Friends	0.613–0.616	1.627–1.632
MSPSS Family	0.536–0.545	1.836–1.867
MSPSS Significant Other	0.494–0.504	1.984–2.025
Partner encouragement	0.946–0.968	1.032–1.057
